# Supplementary material for: Safety and Cross-Neutralizing Immunity Against SARS-CoV-2 Omicron Sub-Variant After a Booster Dose with SOBERANA® Plus in Children and Adolescents
Source: Vaccines (Basel). 2025 Nov 27;13(12):1198. doi: 10.3390/vaccines13121198 (PMC12737616; doi:10.3390/vaccines13121198)
Supplement: Supplementary file 1 [file vaccines-13-01198-s001.zip › vaccines-3917175-supplementary.pdf]

S1. Immunogenicity Assessment and Techniques

S1.1. Anti-RBD IgG response

Anti-RBD IgG in sera was evaluated by a quantitative ultramicro ELISA kit (UMELISA SARS-CoV-2 anti- RBD UM 2045/2145, Centre for Immunoassay, Havana, Cuba) using RBD as coating antigen (4 μg/mL) and an in-house standard characterized serum, which was arbitrarily assigned 200 AU/mL (based on a half-maximal inhibitory titer of 200 and a conventional virus neutralization titer of 160). The standard curve comprised two-fold serial dilutions (0, 4, 8, 16, 32 and 64 AU/mL) of the standard. Samples were evaluated in duplicate. After incubation, biotin-conjugate anti-IgG human (0.1 μg/mL) (Cat# B3773, Sigma Aldrich, San Luis, USA) and then, streptavidin/alkaline-phosphatase (Cat#10556602103, Roche, Basel, Swiss) in appropriate buffer were added. The final fluorometric reaction was induced by adding the substrate 4-Methylumbelliferyl Phosphate (Cat#M3168, Sigma Aldrich, San Luis, USA). The reference curve was constructed using a linear interpolation function. The concentration of anti-RBD IgG was expressed as AU/mL; negative samples were reported as 1.95 AU/mL; samples over 7.8 AU/mL were positive.

S1.2 Inhibitory capacity of antibodies for blocking the RBD-hACE2 interaction

A competitive ELISA determined the inhibitory capacity of antibodies for blocking the RBD-hACE2 interaction. It was expressed as % inhibition and molecular virus neutralization titer ($mVNT_{50}$). Microtiter plates were coated with 250 ng/well of ACE2-hFc in carbonate-bicarbonate buffer, 0.1M (pH 9.6) and incubated overnight at 4°C. Plates were blocked with 200μL/well of 2% of skim milk in PBST (PBS with Tween 20 0.05%) during 1 h, at 37°C. Serial dilutions of sera were pre-incubated with RBD-mouse-Fc (RBD-mFc) at a final concentration of 20 ng/mL, for 1 h at 37°C. These mixtures were added to the plates and incubated for 2 h at 37°C. The binding of RBD-mFc was detected by addition of alkaline phosphatase-conjugated anti-mouse IgG antibody (Cat# A9316, Sigma Aldrich, San Luis, USA) for 1 h at 37°C. Finally, p-nitrophenylphosphate (Sigma Aldrich, San Luis, USA) at 1 mg/mL in diethanolamine buffer (pH 9.8) was added, and plates were incubated at RT for 30 min. The OD at 405nm was measured using a microwell system reader (BioTek). In all steps other than blockade, samples and reagents were added to a final volume of 50 μL/well. Three washing steps with PBST followed all incubations. RBD-mFc, sera and antibody conjugates were diluted in skim milk 0.2%/PBST. Inhibition was expressed as percentage according to the formula: Inhibition (%) = [1-(OD_{405nm} sample/OD_{405nm} maximal recognition)] x 100. Maximal recognition corresponds to wells incubated only with RBD-mFc (20 ng/mL). For determination of $mVNT_{50}$, dilutions were log transformed and the highest dilution giving 50% of inhibition was calculated.

S1.3. Conventional Virus Neutralization titer

This test—the gold standard for determining antibody efficacy against SARS-CoV-2— using live SARS-CoV-2 was performed in a biosecurity laboratory level 3 (National Civil Defense Research Laboratory, Havana, Cuba) by the conventional virus neutralization test. Serial dilutions of heat-inactivated serum samples (starting from 1:5) in Eagle's Minimal Essential Medium (Gibco, UK) containing 2 % fetal bovine serum (Capricorn, Germany) were incubated for 1 hour at 37°C with an equal volume of viral solution containing 100 $TCID_{50}$ of SARS-CoV-2 (strains: CU2010-2025, variant D614G; DC-RRR/2201, variant BA1.21K Omicron; from Cuban Collection at National Civil Defense Research Laboratory) in cell plates containing a semiconfluent VeroE6 monolayer (10^4 cell/well, ATCC Cat# CRL-1586). The highest serum dilution, showing an OD at 540 nm representing 50% of average OD values from control cell wells (VeroE6 monolayer with a mixture of virus-serum) was considered as the neutralization titer and is represented as conventional virus neutralization titer 50 ($cVNT_{50}$).

Table S1. Global characterization of adverse events after the booster dose with SOBERANA® Plus vaccine

	**Age group**		
	**3–11 y/o**	**12–18 y/o**	**Total**
**Total adverse events**	50	38	88
**Intensity**			
Mild	49 (98.0%)	37 (97.4%)	86 (97.7%)
Moderate	1 (2.0%)	1 (2.6%)	2 (2.3%)
**Severity**			
Not serious	50 (100%)	38 (100%)	88 (100%)
Serious	0 (0.0%)	0 (0.0%)	0 (0.0%)
**Causality**			
Consistent (A1)	46 (92.0%)	36 (94.7%)	82 (93.2%)
Undetermined (B1)	1 (2.0%)	0	1 (1.1%)
Inconsistent (C)	3 (6.0%)	2 (5.3%)	5 (5.7%)
**Result**			
Recovered	50 (100%)	38 (100%)	88 (100%)
**Type**			
Local	42 (84.0%)	33 (86.8%)	75 (85.2%)
Systemic	8 (16.0%)	5 (13.2%)	13 (14.8%)
**Solicited**			
Solicited	45 (90.0%)	33 (86.8%)	78 (88.6%)
Unsolicited	5 (10.0%)	5 (13.2%)	10 (11.4%)
**Starts at**			
0-3 hours	17 (34.0%)	13 (34.2%)	30 (34.1%)
3-24 hours	21 (42.0%)	18 (47.4%)	39 (44.3%)
24-48 hours	10 (20.0%)	5 (13.2%)	15 (17.0%)
48-72 hours	0 (0.0%)	1 (2.6%)	1 (1.1%)
> 72 hours	2 (4.0%)	1 (2.6%)	3 (3.4%)
**Duration (hours)**			
0-3 hours	6 (12.0%)	3 (7.9%)	9 (10.2%)
3-24 hours	7 (14.0%)	12 (31.6%)	19 (21.6%)
24-48 hours	21 (42.0%)	19 (50.0%)	40 (45.5%)
48-72 hours	5 (10.0%)	3 (7.9%)	8 (9.1%)
> 72 hours	11 (22.0%)	1 (2.6%)	12 (13.6%)

Table S2. Frequency of solicited adverse events after the booster dose with SOBERANA® Plus vaccine

	Age group		
	**3–11 y/o**	**12–18 y/o**	**Total**
n	129	115	244
**Subjects with some AE**	22 (17.1%)	22 (19.1%)	44 (18.0%)
**Subjects with some solicited local AE**			
Any	19 (14.7%)	21 (18.3%)	40 (16.4%)
Local pain	11 (8.5%)	15 (13.0%)	26 (10.7%)
Swelling	10 (7.8%)	6 (5.2%)	16 (6.6%)
Local warm	9 (7.0%)	5 (4.3%)	14 (5.7%)
Erythema	9 (7.0%)	6 (5.2%)	15 (6.1%)
Induration	3 (2.3%)	1 (0.9 %)	4 (1.6%)
**Subjects with some systemic solicited local AE**			
Any	3 (2.3%)	0 (0.0%)	3 (1.2%)
Fever (≥38 °C)	2 (1.6%)	0 (0.0%)	2 (0.8%)
Low grade fever (<38 °C)	1 (0.8%)	0 (0.0%)	1 (0.4%)

AE= Adverse Event

Table S3. Unsolicited adverse events after the booster dose with SOBERANA® Plus vaccine

	Age group		
	**3–11 y/o**	**12–18 y/o**	**Total**
***n* (%)**	129	115	244
**Number of subjects with unsolicited AE**	5 (3.9%)	4 (3.5%)	9 (3.7%)
**Consistent with vaccination**	1 (0.8%)	3 (2.6%)	4 (1.6%)
Headache	0 (0.0%)	1 (0.9 %)	1 (0.4%)
Functional impotence of arm	0 (0.0%)	1 (0.9 %)	1 (0.4%)
Pruritus at the injection site	1 (0.8%)	1 (0.9%)	2 (0.8%)
**Not consistent**	3 (2.3%)	2 (1.7%)	5 (2.0%)
Acute respiratory infection	1 (0.8%)	2 (1.7%)	3 (1.2%)
Acute intake	1 (0.8%)	0 (0.0%)	1 (0.4%)
Febrile syndrome	1 (0.8%)	0 (0.0%)	1 (0.4%)
**Undetermined (B1)**	1 (0.8%)	0 (0.0%)	1 (0.4%)
Arrhythmia	1 (0.8%)	0 (0.0%)	1 (0.4%)

Table S4. Antibody response before and 28 days after booster dose with SOBERANA® Plus vaccine. in children classified as protein N-negative or positive.

	Age group 3–18 years-old children			
	Protein N negative		Protein N positive	
	**Pre-booster**	**Pre-booster**	**Pre-booster**	**Pre-booster**
n	61	61	156	156
**IgG anti-RBD** median AU/mL	87.4	1062.4**	231.6	791.0**
25-75th percentile	37.4; 178.5	421.6; 1867.2	106.9; 489.8	488.2; 1465.6
**mVNT50** GMT	451.5	8788.4**	1541.2	7009.7**
[CI 95%]	289.6; 703.9	6979.5; 11066.2	1275.8; 1861.7	6055.2; 8114.6
**N**	36	36	43	43
**cVNT50 vs D614G** GMT	60.2	1487.4**	435.0	1354.4**
[CI 95%]	38.4; 94.4	1142.5; 1936.5	299.1; 632.6	1031.8; 1778.0
**N**	26	26	26	26
**cVNT50 vs Omicron BA.1** GMT	35.0	1141.4**	253.0	920.9**
[CI 95%]	20.2; 60.8	721.9; 1804.7	97.9; 238.9	598.5; 1417.1

IgG anti-RBD: Concentration of IgG anti-RBD antibodies, AU/mL= arbitrary units/mL mVNT50: Molecular neutralization titer. cVNT50: Viral neutralization titer.
MGT= Media Geometric titer. CI 95%= Confidence Interval 95%; 25–75th= percentile 25–75th.
Wilcoxon Signed-Rank Test were used for before-after statistical comparison and Mann-Whitney U test for N+ vs N- comparison. **p<0.05; **p<0.005
